# UNDERSTANDING CHALLENGES AND SOLUTIONS FOR DEAF OLDER ADULTS

**DOI:** 10.1093/geroni/igad104.3680

**Published:** 2023-12-21

**Authors:** Shraddha Shende, Lyndsie Koon, Jenny Singleton, Wendy Rogers

**Affiliations:** Illinois State University, Normal, Illinois, United States; Kansas University, Lawrence, Kansas, United States; Stony Brook University, Stony Brook, New York, United States; University of Illinois Urbana-Champaign, Champaign, Illinois, United States

## Abstract

The Aging Concerns, Challenges, and Everyday Solution Strategies (ACCESS) project explores everyday activity challenges and solutions experienced by U.S. older adults as they age with a lifelong disability involving vision, motor, or hearing. Here, we report our findings from our community-engaged research with older adults who self-identify as members of the U.S. Deaf Community and who use American Sign Language (ASL) as their primary language. Using trained Deaf interviewers, we conducted semi-structured interviews in ASL with 60 Deaf participants (age 60-79 years) to understand their challenges performing various everyday activities (e.g., doing things around the home, activities outside the home, transportation, and managing health). Overall, our thematic analyses revealed that Deaf older adults navigate everyday tasks quite well. However, they experience particular challenges with technology, communication, and accessibility; specifically, fragile or inaccessible technology systems. For instance, technology alerting systems at home (e.g., smoke alarms) or airports (e.g., gate change announcements) are typically auditory. Accessibility is also poor when healthcare or wellness providers fail to provide an ASL interpreter. When probed about their responses to these challenges, Deaf older adults often reported frustration or resignation when experiencing barriers; the need for constant vigilance and self-advocacy; or reliance on hearing family members when systems fail them. Improving communication access and technologies that Deaf older adults rely on is critical and requires the inclusion of individuals from the Deaf community to identify the challenges and potential avenues for solutions.

